# Does food insecurity link to suicidal behaviors among in-school adolescents? Findings from the low-income country of sub-Saharan Africa

**DOI:** 10.1186/s12888-019-2212-6

**Published:** 2019-07-24

**Authors:** Festo K. Shayo, Paul S. Lawala

**Affiliations:** 1 Department of Internal Medicine, Muhimbili University of Health and Allied Sciences, P. O Box 65001, Dar es Salaam, Tanzania; 2 Department of Psychiatry and Mental Health, Mbeya Zonal Referral Hospital, P.O. Box 419, Mbeya, Tanzania; 3 Department of Global Health Entrepreneurship, Division of Public Health, Graduate School of Tokyo Medical and Dental University, 1-5-45 Yushima, Bunkyo-ku, Tokyo, 113-8510 Japan

**Keywords:** Adolescents, Suicide behaviors, Food insecurity, A low-income country, Sub-Saharan Africa

## Abstract

**Background:**

Suicidal behaviors among adolescents is a major public health concern. Psychological factors have been extensively studied known risks linked to suicidal behaviors in the general population. However, the association between food insecurity and suicidal behaviors is less researched, particularly in low and middle-income countries. The present study sought to assess the link between food insecurity and suicide behaviors among in-school adolescents.

**Methods:**

The present study was based on the secondary analysis of the first nationally representative sample of the 2014 Tanzania Global School-based Student Health Survey (GSHS). A total sample of 3,793 in-school adolescents was included in the present analysis. The primary independent variable was food insecurity while the dependent variables of interest were suicide ideation and suicide attempt. Chi-square χ^2^ and multivariate logistic regression were used to ascertain the measure of statistical association. In all analyses, a *p* < 0.05 was considered statistically significant.

**Results:**

Of the 3,793 in-school adolescents, 254 (6·7%) were food insecure. A significantly large proportion of adolescents with suicidal ideation and suicidal attempt were food insecure than their counterparts, respectively. In the adjusted multivariate model, food insecure adolescents were more likely to have experienced suicidal ideation and suicidal attempt: [AOR; 1·8 95% C. I; 1·3–2·5] and [AOR; 2·4, 95% C. I; 1·7–3·3]; *p* < 0.001, respectively.

**Conclusion:**

Food insecurity was an independent predictor of suicidal behaviors among in-school adolescents. An intervention that targets food security at the school level may protect adolescents of food insecure household from suicidal behaviors. Nevertheless, school-based mental health screening, evaluation, and promotion may be needed for adolescents with suicidal behaviors.

## Background

Globally every year about 800,000 people die of suicide, which represents the age-standardized rate of 11·4 per 100,000 populations [1]. The mortality rate is slightly higher in high-income countries than low and middle-income countries (LMICs); 12·7 versus 11·2 per 100 000 populations. However, 75·5% of all global suicide occur in LMICs where a large proportion of the world population resides [2]. In 2015 suicide was the second cause of deaths among adolescents and young adults of age group 15–29 years [2, 3]. Nonetheless, the global and across individual countries’ suicide rate may be underestimated because suicide is a sensitive incident less discussed openly in society, and it is an illegal act in some countries [1]. Globally, few countries have included suicide prevention in their health priority agendas and only 28 countries are reported to have a national suicide prevention strategy [1, 4].

In Tanzania, the overall age-standardized suicide rate per 100,000 was 24·9 in the year 2012, higher than the global average rate, 11.4. Moreover, it was found that the rate was 3·5 and 20.7 in the age groups 5–14 yrs., and 15–29 yrs., respectively [1].

Suicide behaviors constitute; suicide ideation (thinking about killing oneself), suicide plan (planning on killing oneself), suicide attempt (attempting to kill oneself), and suicide itself (committing suicide or killing oneself) of which a suicide attempt is the most single strongest predictor of suicide in the general population [1]. The epidemiology of suicidal behavior has been reported by a number of studies in European countries and across 49 LMICs. For instance, the lifetime prevalence of suicidal ideation among European school adolescents aged 15–16 years were reported to range from 15% in Armenia to 31.5% in Hungary, while that of suicide attempts ranged from 4.1 to 23.5% in the same two countries, respectively [2]. In a review across 49 LMICs, 15.3% of the adolescents aged 13–15 years were reported to have been seriously considered suicide in 1 year preceding the surveys [5]. In Tanzania, the prevalence of suicidal behaviors among school-attending adolescents was reported in a study conducted in Dar es Salaam city in 2006. Among surveyed students aged 11–16 years; 7% reported suicidal ideation, and 6.3% suicidal plan. Psychological health (loneliness and depressive symptoms), substance use, and being bullied were factors significantly associated with suicidal behaviours [6].

Among the common explored risk factors for suicide behaviors in adolescents are psychosocial factors; loneliness, anxiety, and lack of parental support [1, 7, 8]. However, food insecurity is less explored as a risk factor for suicidal behaviors in the general population including adolescents.

Food insecurity exists when people do not have adequate physical, social or economic access to sufficient, safe and nutritious food which meets their dietary needs and food preferences for the active and healthy life [9, 10]. On the other hand, hunger is a physiological condition caused by severe food insecurity. All hungry people are food insecure, but not all food insecure people are hungry [10]. The global and regional prevalence of severe food insecurity has been shown to increase. For instance, the global prevalence was 8.9 and 10.2%, while in sub-Saharan Africa region was 25.0 and 33.8% in the year 2014 and 2017, respectively [9].

The mechanism that links between food insecurity and suicidal behaviors has been explained by a number of theories including some few studies. Potential biological and stress mechanisms have been suggested to be responsible for the association between food insecurity and poor mental health such as suicidal behaviors [11]. Food insecurity could be a source of embarrassment, anxiety, and stress [12], which may be responsible for the exacerbation of mental disorders. The presence of poor mental health among food insecure individuals has been reported among various risk populations [13–16].

A limited number of studies in high-income countries have reported the association between food insecurity and poor mental health among in-school adolescents. For instance, studies in Canada and the United States of America (USA) revealed that food insecure adolescents were significantly more likely to have had experienced suicide ideation, attempt, and depressive disorders [17, 18]. Similarly, one study in Southeast Asia revealed that adolescents who are food insecure were more likely to have had experienced suicide ideation and attempt [19].

Mental health disorders, including psychosocial factors and substance abuse, have been extensively studied and found to be the main risk factors for suicidal behaviours [20]. School-based studies in LMICs have reported the association between suicidal behaviors and psychosocial factors among in-school adolescents. For instance, studies from two counties of Sub-Saharan Africa; Seychelles and Benin, revealed that in-school adolescents suffering from anxiety and loneliness were more likely to have had experienced suicidal ideation and attempt. On the other hand, a good parent-child attachment was shown to be a protective factor against suicidal behaviour [21, 22]. Similarly, studies in Southeast Asia and the Middle East revealed that in-school adolescents with suicide ideation and the attempt were more likely have had experienced anxiety, loneliness, and poor parental attachment [19, 23, 24].

In Tanzania, the prevalence of suicide behaviors and the associated risk factors among in-school adolescents was reported in one study conducted in Dar es Salaam region in the year 2006. The study found that the suicide behaviors among in-school adolescents were significantly associated with psychosocial factors including; anxiety, loneliness, and poor parent-child attachment [6]. However, no study has yet reported the link between food insecurity and suicidal behaviors among adolescents in sub-Saharan Africa, including Tanzania. Therefore, the main aim of the present study was to assess the link between food insecurity and suicide behaviors among in-school adolescents besides the common known psychosocial factors.

## Method

### Data source

The present study is based on a secondary analysis of the first nationwide representative sample of the 2014 Tanzania Global School-based Student Health Survey (GSHS). The dataset used for analysis in the present study is free available in the following WHO repository website: http://www.who.int/ncds/surveillance/gshs/tanzaniadataset/en/.

### Sampling technique and sample size

By adhering to the GSHS methodology, students were selected using a two-stage cluster sampling. The aim was to produce a nationwide representative sample of students from both primary schools (grades/standards 6–7) and secondary schools (form 1–3) aged 13–17 years. At the first stage of sampling, schools were selected with the probability proportional to their reported enrolment size in the national sampling frame. In total, 50 schools out of 20,230 schools were randomly selected to participate in the Tanzania GSHS. In the second stage, systematic random sampling was used to select classrooms from each selected school. All students in the selected classrooms were eligible to participate in the study regardless of their actual age. The school and student response rate was 100 and 87%, respectively. A total of 3,793 students participated and completed the study survey.

### Data management

The 2014 Tanzania GSHS, used a standardized questionnaire of the WHO GSHS to develop a country-based questionnaire. The questionnaire was translated into the Swahili language before pre-tested and used for the survey. Students who consented to participate in the survey were given a self-administered questionnaire. In order to protect the students’ privacy, the survey adhered to the principles of anonymous and voluntary participation. The survey used a weighing factor to each student to adjust for non-response and the varying probability of selection. The 2014 GSHS Tanzania was approved by the Tanzania Ministry of Health, Community Development, Gender, Elderly and Children (MoHCDGEC); and the Ministry of Education, Science and Technology (MoEST).

### Operational definitions of variables

Food insecurity exists when people do not have adequate physical, social or economic access to sufficient, safe and nutritious food which meets their dietary needs and food preferences for an active and healthy life. All hungry people are food insecure, but not all food insecure people are hungry [10]. For the purpose of the present study, food insecurity is defined as whether the student went hungry due to a shortage of food in the home over the past 30 days preceding the survey.

On the other hand, Suicide behaviors constitute; suicide ideation (thinking about killing oneself), suicide plan (planning on killing oneself), suicide attempt (attempting to kill oneself), and suicide itself (committing suicide or killing oneself) [25]. For the purpose of the present study, suicide behaviors refer to suicide ideation and attempt.

### Dependent variables

The primary dependent variables were suicide ideation and suicide attempt. These dependent variables were taken from the Tanzania GSHS survey public use codebook questionnaire. Regarding suicide ideation, the following question was asked: During the past 12 months, did you ever seriously consider attempting suicide? Responses were “Yes” coded as 1 and, “No” coded as 2. Regarding the suicide attempt, the following question was asked: During the past 12 months, how many times did you actually attempt suicide? The response was “Yes” for 1,2,3,4,5, and ≥ 6 times attempt coded as 1 and, “No” for zero attempts coded as 2.

### Independent variables

#### Primary independent variable

Food insecurity: During the past 30 days, how often did you go hungry because there was not enough food in your home? The responses were never, rarely, sometimes, most of the time and always. The responses were “Yes” (combined most of the time and always) and, “No” (combined never, rarely and sometimes). For easy analysis, the original question was created into a dichotomous variable (according to GSHS Data User’s Guide); “Percentage of students who most of the time or always went hungry (because there was not enough food in their home during the 30 days before the survey)”. The responses were “Yes” coded as 1, and “No” coded as 2.

#### Other independent variables

Loneliness: During the past 12 months, how often have felt lonely? The responses were never, rarely, sometimes, most of the time, and always. The responses were coded into 1 for “Yes” (combined most of the time and always) or 2 for “No” (never, rarely and sometimes).

Anxiety: During the past 12 months, how often have you been so worried about something that you could not sleep at night? The responses were coded into 1 for “Yes” (combined most of the time and always) or 2 for “No” (never, rarely and sometimes).

Parent-child attachment: During the past 30 days, how often did your parents or guardians understand your problems and worries? The response options were never, rarely, sometimes, most of the time, and always. The responses were “Yes” (combined most of the time and always) and, “No” (combined never, rarely and sometimes). For easy analysis, the original question was created into a dichotomous variable (according to GSHS Data User’s Guide); “ Percentage of students who reported that their parents or guardians most of the time or always understood their problems and worries (during the 30 days before the survey)”. The responses were “Yes” coded as 1, and “No” coded as 2.

The other variables were participant’s demographics; gender (male and female), age (categorized into ≤12, 13–17 yrs., and > 17 yrs), and school level (categorized into primary and secondary school).

#### Data process and analysis

Since nonresponses were observed to be missing at random (MAR) pattern, a multiple imputation was used to examine the possibility of bias, however, no bias was observed. Replacing the missing data with imputed data did not make differences in outcomes of interest, hence a significant measure of association in both the bivariate and multivariate analyses was maintained despite imputation. A full Conditional Specification (FCS) method was used after automatic command scanned for the data which were missing at random. A maximum of five imputations was run to allow for > 97% efficiency. Multiple imputation is a useful statistic technique replaces each missing value by two or more plausible values of nonresponse in public-use files. Multiple imputation is a useful statistic technique that replaces each missing value by two or more plausible values. Multiple imputations were used in the current study due to the following reasons; first, the pattern of missing data was missing at random [26–28], second, to account for the consistency of the dedicated sample size values of each variable throughout the analyses. Nevertheless, previous studies elsewhere have used a multiple imputation procedure to account for missing data at random [29–31].

Nevertheless, we assessed for multi-collinearity of independent variables with variance inflation factor (VIF) and tolerance. Regarding age and academic level variable, the values for VIF and tolerance were 1.0 and 1.0, respectively. For the variables; loneliness, anxiety, suicide ideation, and suicide attempt the values for VIF and tolerance were 0.982 and 1.112, respectively. Therefore, this signify that no multi-collinearity between the variables as it has been recommended that the critical value for VIF should not exceed 4.

We used both descriptive and inferential statistics in our current analysis. Chi-square χ^2^ test was used to compare the difference between the group variables. All variables with a statistical significance of *p* < 0·05 (except for gender variable) in bivariate analysis were subjected to the multivariate model analysis. An unadjusted and adjusted multivariate logistic regression was fitted to explain the association between food insecurity and suicide behaviors. In all analyses, a *p*-value of less than 0.05 was considered statistically significant. SPSS version 22 (SPSS, Chicago, IL) was used for analysis.

## Results

### Participant’s demographics, the prevalence of suicidal behaviors, and risk factors

Table 1 represents the participants’ demographic characteristics, the prevalence of suicidal behaviours, and risk factors. Of 3,793 in-school adolescents 1,974 (52·1%) were females, 2,987 (78·7%) were aged 13–17 yrs., and 2,085 (55·0%) were in a primary school level (grade 6 and 7).

**Table 1:** Baseline characteristics of the study participants [*N* = 3,793]

Variables	Categories	n[%]
Gender	Male	1819 [47·9]
	Female	1974 [52·1]
Age	≤12 yrs.	681 [18·0]
	13–17 yrs.	2987 [78·7]
	> 17 yrs.	125 [3·3]
School level	Primary school	2085 [55·0]
	Secondary school	1703 [44·9]
	Other grade	5 [0·1]
Food insecurity	Yes	254 [6·7]
	No	3539 [93·3]
Loneliness	Yes	283 [7·5]
	No	3510 [92·5]
Anxiety	Yes	237 [6·2]
	No	3556 [93·8]
Suicide ideation	Yes	536 [14·1]
	No	3257 [85·9]
Suicide attempt	Yes	422 [11·1]
	No	3371 [88·9]
Parent-child attachment	Yes	1455 [38·4]
	No	2338 [61·6]

The prevalence of food insecurity was 254 (6·7%) while 536 (14·1%), and 422 (11·1%) experienced suicide ideation and suicide attempt, respectively in the past 12 months preceding the survey.

### Risk factors and suicide behaviors

Table 2 represents the comparison of the risk factors and demographics with suicide behaviors. A significantly large proportion of adolescents who reported suicide attempt compared to their counterparts; were food insecure 15·9% vs 5·6%, experienced loneliness 17·5% vs 6·2%, experienced anxiety 18·7% vs 4·7%, and lacked parental attachment 66·7% vs 60·6%. Similarly, a significantly large proportion of adolescents who reported suicide ideation were food insecure, experienced loneliness and anxiety, and lacked parental attachment.

**Table 2:** Risk factors for suicide behaviors among in-school adolescents (*N* = 3,793)

Variables		Suicide ideation			Suicide attempt		
	Categories	Yes n[%]	No n [%]	*P* value	Yes n [%]	No n [%]	*P* value
Gender	Male	271 [50·5]	1548 [47·5]	0.35	192 [45·4]	1627 [48·2]	0.3
	Female	265 [49·5]	1709 (52·5)		230 [54·6]	1744 [51·8]	
Age	≤12 yrs.	150 [27·6]	531 [16·4]	P < 0.001	110 [25·6]	571 [17·0]	P < 0.001
	13–17 yrs.	380 [69·8]	2607 [80·2]		311 [72·3]	2676 [79·6]	
	> 17 yrs.	14 [2·6]	111 [3·4]		9 [2·1]	116 [3·4]	
School level ^a^*N* = 3748	Primary	315 [59·7]	1750 [54·3]	P < 0.05	262 [63·1]	1803 [54·1]	P < 0.001
	Secondary	213 [40·3]	1470 [45·7]		153 [36·9]	1530 [45·9]	
Food insecurity	Yes	61 [11·4]	193 [5·9]	P < 0.001	67 [15·9]	188 [5·6]	P < 0.001
	No	475 [88·6]	3064 [94·1]		355 [84·1]	3183 [94·4]	
Loneliness	Yes	63 [11·7]	220 [6·8]	P < 0.001	74 [17·5]	209 [6·2]	P < 0.001
	No	474 [88·3]	3036 [93·2]		348 [82·5]	3162 [93·8]	
Anxiety	Yes	65 [12·1]	172 [5·3]	P < 0.001	79 [18·7]	158 [4·7]	P < 0.001
	No	471 [87·9]	3085 [94·7]		343 [81·3]	3213 [95·3]	
Parent-child attachment	Yes	174 [32·5]	1281 [39·3]	*P* < 0.01	128 [33·3]	1327 [39·4]	P < 0.001
	No	362 [67·5]	1976 [60·7]		294 [66·7]	2044 [60·6]	

Note: ^a^ A category; other grades were not included

### Predictors of suicidal behaviors

#### Food insecurity and suicide behaviors

After adjusting for all other variables in the model, suicidal ideation and the attempt were significantly associated with food insecurity. Adolescents who were food insecure in the past 30 days preceding the survey were significantly more likely to have experienced suicide ideation [AOR; 1·8 95% C. I; 1·3–2·5] and suicide attempt [AOR; 2·4, 95% C. I; 1·7–3·3]. For more details, refer to Table 3.

**Table 3:** A crude and adjusted multivariate logistic regressions of the factors associated with suicidal behaviors among in-school adolescents (*N* = 3,793)

Variables		Suicide ideation		Suicide attempt	
		OR [95% C.I]	AOR [95% C.I]	OR [95% C.I]	AOR (95% C.I)
Food Insecurity (Ref. No)	Yes	2·1 [1·5–2·9] *P* < 0.001	1·8 [1·3–2·5] *P* < 0.001	3·2 [2·3–4·3] *P* < 0.001	2·4 [1·7–3·3] P < 0.001
Loneliness (Ref. No)	Yes	1·9 [1·4–2·5] P < 0.001	1·5 [1·1–2·1] *P* = 0.02	3·2 [2·4–4·3] *P* < 0.001	2·2 [1·6–3·0] P < 0.001
Anxiety (Ref. No)	Yes	2·5 [1·8–3·4] *P* < 0.001	2·1 [1·5–2·9] *P* < 0.001	4·5 [3·4–6·1] P < 0.001	3·4 [2·4–4·7] P < 0.001
Parental care (Ref. No)	Yes	0·7 [0·6–0·9] *P* = 0.004	0·8 [0·6–0·9] *P* = 0.02	0·7 [0·5–0·9] P < 0.001	0·7 [0·6–0·9] *P* = 0.002
^a^AGE		0·5 [0·4–0·7] P < 0.001	0·5 [0·4–0·6] P < 0.001	0·7 [0·5–0·8] P < 0.001	0·7 [0·5–0·9] *P* = 0.002
Gender (Ref. Female)	Male	1·1 [0·9–1·4] *P* = 0.15	1·2 [1·0–1·5] *P* = 0.027	0·9 [0·7–1·1] P = 0·31	0.9 [0·8–1·2] P = 0·48
School level (Ref. Secondary)	Primary	1·2 [1·0–1·5] P = 0.02	0·9 [0·7–1·1] P = 0·3	1·4 [1·2–1·8] *P* = 0.001	1·1 [0·9–1·4] P = 0·32

Note: Food insecurity is adjusted for all independent variables in the model, ^a^Age is included as a continuous variable

#### Psychosocial behaviors and suicidal behaviors

In the adjusted multivariate model, adolescents who reported to be lonely were significantly more likely to have experienced suicidal ideation and attempt; [AOR; 1·5, 95% C. I; 1·1–2·1], and [AOR; 2·2, 95% C. I; 1·6–3·0], respectively, compared to their counterparts. Likewise, those who had anxiety were significantly more likely to have experienced suicidal ideation and attempts; [AOR; 2·1, 95% C. I; 1·5–2·9], and [AOR; 3·4, 95% C. I; 2·4–4·7], respectively. On the other hand, adolescents who had parental attachment were significantly less likely to have experienced suicidal ideation and attempt; [AOR; 0·8, 95% C. I; 0·6–0·9], and [AOR; 0·7, 95% C. I; 0·6–0·9], respectively. For more details, see Table 3.

#### Demographics and suicide behaviors

In the adjusted model, the risk of suicidal ideation and attempt decreases with increasing age; [AOR; 0·5, 95% C. I; 0·4–0·6], and [AOR; 0·7, 95% C. I; 0·5–0·9], respectively. For more details, refer to Table 3.

## Discussion

Suicidal behavior among adolescents is a public health concern, however, its link with food insecurity is less explored. The present study analysis has examined the association between food insecurity and suicide behaviors among in-school adolescents besides other common explored psychosocial risk factors.

The present study found that the prevalence of food insecurity among in-school adolescents was 6.7%. This is the proportion of adolescents who reported to be hungry because there was not enough food in your home. This prevalence is higher than the previous study in Thailand [19] while lower than the study in the Republic of Seychelles [22]. The current situation of food insecurity among in-school adolescents in Tanzania is unclear, however, it might have changed because of the gradual implementation of 2014 education and training policy. For instance, during the year 2014 when this survey was conducted the existed policy stipulated that parents should contribute a certain amount of money for food-related expenses in order to cover the cost of lunch for pupils. However, the contribution amount varied somewhat across the schools, hence disparities in terms of food security at the school level [32]. In 2016 the government abolished all fees, including additional financial requirements for lower secondary education [33]. Therefore, the current scope of food security in public primary and secondary schools in Tanzania is blurred. In the present study, adolescents reported being food insecure merely from their homes. Theretofore, they might have been in access to food at school during the 2014 survey when the education and training policy was still favoring parents to contribute to the food-related cost.

Importantly the present study found the significant association between food insecurity and suicide behaviors among in-school adolescents. Food insecure adolescents were significantly more likely to have been experiencing suicidal ideation and attempts in the past 12 months preceding the survey. This is a new finding in Tanzania context as an example of a low-income country of sub-Saharan Africa. The present study finding is comparable to previous studies in Thailand and Benin [19, 21] as well as studies in high-income countries of the USA and Canada [17, 18].

Food insecurity in children is reported to be linked with a higher rate of a wide range of the adolescent’s mood and behaviors and can lead to depressive symptoms later in life [34]. The mechanism that links between food insecurity and suicidal behaviors has been explained by a number of theories including some few studies. Potential biological and stress mechanisms have been suggested to be responsible for the link between food insecurity and poor mental health such as suicidal behaviors [11]. Food insecurity could be a source of embarrassment, anxiety, and stress [12], which may be responsible for the exacerbation of mental disorders. The presence of poor mental health among food insecure individuals has been reported among various risk populations [13–16]. Despite whatever preexisting mental health, the presence of difficult life events has been directly associated with suicidal ideation [35]. The amount of literature on dieting and starvation has hypothesized that malnutrition and micronutrient deficiency among food insecure populations could explain the link existing between suicide behaviors and food insecurity [36]. More research evidence is needed to further explore the mechanism linking food insecurity and suicidal behaviors besides the commonly known risk factors; psychological factors.

Psychological factors have been extensively explored as predictors for suicidal behaviors. The present study found that psychosocial stresses; loneliness and anxiety to be significantly associated with suicide behaviors among in-school adolescents. Adolescents who were lonely and worried most of the time (anxiety) during the past 12 months preceding the survey were more likely to have experienced suicide ideation and attempt. The present study finding is comparable to previous studies [8, 19, 23, 24]. Psychological disorders have been reported to be present up to about 90% of adolescents who attempted or committed suicide. For instance, in a Finish longitudinal survey anxiety disorders was found to be the strongest predictors of committing and/or attempting suicide among children [7]. Therefore, there is a high need to screen for psychological disorders among in-school adolescents who present with bizarre mental problems or suicidal behaviors.

Family factors such as poor quality parent-childrelationship are associated with an increased risk of suicidal behaviors among adolescents. The present study found that adolescents who had a good parent attachment (parents or guardian who could understand their problems most of the time or always) were significantly less likely to have experienced suicidal ideation and attempt. This finding is comparable to previous studies in Thailand, Benin, and Seychelles [19, 21–23]. In Tanzania, there is one small-scale survey (2006 GSHS) which was done in Dar es Salaam city. In this survey, it was found that in-school adolescents who had good parental attachment were less likely to have experienced suicidal ideation. However, this survey was designed to be a nationally representative until the latest GSHS of the year 2014 [6]. Social and family support may be a protective factor against health risk behaviors such as suicide behaviors among adolescents. There is evidence that school-based education programs for parents of the students can improve family and school protective factors against adolescent’s suicidal behaviors [37].

Furthermore, the present study revealed that the risk of suicidal ideation and attempt decreases with increasing age, even after controlling for all other confounders. This finding is comparable with previous studies in Thailand and the Republic of Seychelles [19, 22], however, it is contrary to study in China [37]. There is a possibility of uncovered risk factors responsible for the increased likelihood of suicide behaviors among younger adolescents. However, some studies have reported a higher prevalence of psychiatric disorders like anxiety in younger adolescents, even at the age of 8 years [7]. Therefore, age-specific mental health evaluation and promotion may be necessary among young adolescents.

The current study has the following strengths; first, we think this is the first study in Tanzania and possibly in sub-Saharan Africa to have examined the link between food insecurity and suicidal behaviors among in-school adolescents. Therefore, we think to some extent the present study can be a representative of low-income countries with limited household food security and poor food policy at the school level. Second, this is the first nationally representative sample analysis to explore the risk factors associated with suicidal behaviors among in-school adolescents in Tanzania. Third, we have used multiple imputation to examine the effect of missing data which is a common occurrence for national survey studies.

On the other hand, the present study has the following limitations; the study was based on adolescents who were attending school and therefore the finding may not be generalizable to other adolescents who were out-of-school. For instance, in the year 2014, the gross enrollment of pupils in Tanzanian primary and secondary school was 82.62 and 31.67%, respectively [38]. There were about 2.2 million out-of-school children at the primary school level (aged 7–13), and 1.7 million out-of-school children at the lower secondary school level (aged 14–17) by 2015 [39]. Second, since the questionnaire was self-reported it relied upon study participants recall memory, therefore, there could be potential recall bias. Third, some variable in our study, such as parental-child attachment was based on a single response to a single item concept, hence could have unfairly reflected a complex concept behind parental care and suicidal behaviors among adolescents. Fourth, the current study did not collect data about family factors such as the family history of suicidal behaviors and the number of guardians/parents. Studies have shown that a family history of suicidal behaviors, family discord, loss of a parent to death or divorce are associated with a higher risk of suicidal behaviors among adolescents [7]. The inclusion of these factors in the survey could have strengthened the present study.

## Conclusions

The current study provides valuable insight about the rarely explored phenomenon of the link between food insecurity and suicidal behaviors among adolescents, and particularly of LMICs setting with food insecurity and probably poor food policy at the school level. The study found that adolescents who are food insecure were more likely to have experienced suicide ideation and attempts. The findings from the present study may be utilized to increase awareness about the scope of food insecurity and its link with mental health disorders such as suicidal behaviors. Furthermore, the present study may inform the policymakers and other potential stakeholders to guide the policy and programs that target food security at schools as well as the household/family level. This may help to mitigate the burden of suicide behaviors and other related food insecurity effects among adolescents. Nevertheless, school-based mental health screening, evaluation, and promotion may be needed for adolescents with suicidal behaviors.

## Data Availability

The dataset file used in the present study analysis is publicly freely available on the WHO repository (http://www.who.int/ncds/surveillance/gshs/tanzaniadataset/en/).

## Abbreviations

AOR: Adjusted odds ratio
CDC: Canters for Disease Control
FAO: Food and Agriculture Organization of the United Nations
GSHS: Global School-based Student Health Survey
HPES: Health Promotion and Education Section
IFAD: International Fund for Agricultural Development
LMICs: Low and Middle-income countries
MAR: Missing at random
MoEVT: Ministry of Education and Vocational Training
MoHSW: Ministry of Health and Social Welfare
NSHP: National School Health Program
OR: Odds ratio
UNICEF: United Nations Children’s Fund
USA: United States of America
WFP: World Food Program
WHO: World Health Organization
